# The Effects of Sertraline in Controlling Refractory Hypertension in Women with Premenstrual Syndrome

**Published:** 2016-10

**Authors:** Fatemeh Ranjbar, Fariborz Akbarzadeh, Mahboub Asadlou

**Affiliations:** 1 Clinical Psychiatry Research Center, Tabriz University of Medical Sciences, Tabriz, Iran.; 2 Cardiovascular Research Center, Tabriz University of Medical Sciences, Tabriz, Iran.; 3 Clinical Psychiatry Research Center, Tabriz University of Medical Sciences, Tabriz, Iran.

**Keywords:** *Premenstrual Syndrome*, *Refractory Hypertension*, *Sertraline*

## Abstract

**Objective:** The aim of this study was to evaluate the effect of Premenstrual Syndrome (PMS) treatment with selective serotonin reuptake inhibitor (SSRI) on treatment response of refractory hypertension of the patients.

**Method:** This was a triple-blind randomized clinical trial conducted on female patients suffering from refractory hypertension and PMS at the same time. We obtained informed consent from 40 patients who had inclusion criteria and selected 20 patients for the intervention (sertraline 50 mg daily) and 20 for the control groups. The study period was five weeks. The mean of systolic and diastolic blood pressure before and after intervention was measured separately for each individual in each group and the mean of blood pressure of the members of the two groups were compared with each other.

**Results:** The mean age of the participants was 43.60 ± 4.57. In this study, systolic and diastolic blood pressure of both groups reduced after intervention. The mean of systolic blood pressure was reduced by 40.86 mmHg in the intervention group and this reduction was 16 mm Hg in control group after intervention (P<0.001). Comparing this reduction between the two groups, we found that reduction rate in systolic blood pressure of the two groups did not have a significant statistical difference before and after the intervention (P = 0.11). Mean of diastolic blood pressure also showed reduction of 9.17 mm Hg and that of control group showed 6.7-mmHg reduction. Reduction rate of diastolic blood pressure in the intervention group had a statistically significant difference with that of the control group (P<0.017).‎‎

**Conclusion**: Administration of sertraline is more effective in controlling diastolic blood pressure in women suffering from refractory hypertension and comorbid PMS.

Clinical disorders related to mood and behavior in premenstrual phase have been the focus of attention since the past (1). Premenstrual syndrome (PMS) and its stronger and more specific form, premenstrual dysphoric disorder (PMDD), have been classified as Not Otherwise Specified Disorder (NOS). 

While PMS could affect 80% of women in childbearing age, PMDD incidence is about 5% (2). If symptoms have enough intensity to disturb daily life or interpersonal relations, PMDD would be considered (3). PMDD has research criteria in Diagnostic and Statistical Manual of Mental Disorders (DSM-IV-TR), and its main symptoms are low mood, stress, emotional instability and reduction of interests in activities (4).

Some randomized control trial studies on women suffering from PMDD have shown that selective serotonin reuptake inhibitors (SSRIs) have suitable effects with least side effects (5-9). A systematic review also confirmed that SSRIs and Clomipramine are effective in reducing symptoms of premenstrual Although PMS is not a proven factor for hypertension nor is one of the factors reducing the impact of antihypertensive medications (11), hypertension is higher in patients with PMS (12) and it is suggested that PMS be considered in women with hypertension, whose control of blood pressure is difficult and are not yet in their postmenopausal period (11).

Studies have shown that in women with PMS, activity of the sympathetic nervous system increases in final stages of luteal phase and inversely activity of parasympathetic nervous system decreases in this stage (13, 14). In premenstrual phase, there is a tendency to sodium and fluid retention (2), and in those with severe PMS, activity of parasympathetic system reduces during sleep (15).

The primary objective of treating those suffering from hypertension, is reaching minimum cardiovascular disorders, which is possible through reduction of blood pressure and reversible risk factors (16). No comprehensive study has been conducted on the effect of controlling PMS symptoms with SSRIs and since the prevalence of PMS is high in women in childbearing age and lack of control on women’s refractory hypertension could lead to significant consequences, conducting such a study could be of prime importance.

The aim of this study was to evaluate the effect of PMS treatment with sertraline to control refractory hypertension in women with premenstrual syndrome.

## Materials and Method

This was a triple- blinded randomized controlled study performed from June 2010 to June 2012. In this study, 100 female patients with the age range of 15 to 49 who suffered from refractory hypertension (patients for whom despite lifestyle modification and receiving 3 anti-hypertension drugs, one of which was diuretics, systolic blood pressure lower than 140 or diastolic lower than 90 could not be preserved) and referred to the cardiology clinic of Tabriz University of Medical Sciences, the main referral center in Northwest of Iran, were selected.

Among these 100 refractory hypertension patients, 40 who were also suffering from premenstrual syndrome based on test of Daily Record of Severity of Problems (DRSP) were selected after providing written informed consent. DSRP is a method used for diagnosis and evaluation of DSM-IV Premenstrual Dysphoric Disorder (17).

Using Randlist software (Version 1.2, DatInf GmbH, Tubingen, Germany) patients were divided into two groups randomly: one group received 50 mg sertraline daily and the other received placebo. 

Inclusion criteria were being female, having refractory hypertension, having criteria for premenstrual dysphoric disorder, age between 15 and 49, and providing written informed consent. Exclusion criteria were history of allergy or complication to sertraline, bipolar mood disorders, depressive disorders and anxiety, psychosis, mental retardation, breastfeeding and pregnancy, acute coronary syndrome and heart failure. Patients with psychiatric disorders were excluded from the study, using a structured routine psychiatric interview.

In this study, the physicians, patients and statisticians were not aware of type of consumed drug (medication or placebo). This was a triple- blinded study. Patients used their anti-hypertension medications as usual after joining the study, and no change was made in anti-hypertension drug regimen of the patients. Patients in the intervention group received 50 mg sertraline daily (made in Sobhan Pharmaceutical Company) for five weeks. Patients in the control group underwent treatment by placebo with a similar duration. 

The two groups were studied for five weeks, and between weeks 2 and 3, the patients of both groups were controlled in terms of medication side effects (using a related checklist) in person or by phone; and the checklist of side effects of SSRIs was completed for all patients. In addition, both groups were studied in terms of symptoms of sudden increase or decrease in blood pressure by history taking. TONOPORT

All participants signed a written consent, and the Ethics Committee of Tabriz University of Medical Sciences (TUMS) approved the study protocol, which was in compliance with Helsinki Declaration. The registration code of this study in Iranian Registry of Clinical Trials (IRCT) web site is IRCT138904092181N4.

At the beginning of study and at the end of the fifth week, blood pressure of both groups were controlled again by 24-hour TONOPORT (PAR Medizintechnik GmbH & Co. , Berlin, Germany) , which is a portable patient monitor for ambulatory blood pressure measurement. The mean of blood pressure, minimum and maximum BP and even blood pressure of the patient in nighttime was obtained and all were compared with pre-study blood pressure. 

Obtained data have been represented as Mean ± Standard deviation (Mean ± SD), and also distribution and percentage. SPSSTM Version 15 (SPSS ltd, Chicago, IL, USA) was used for statistical analysis. Independent t-test and chi square test were used to analyze the data. P value less than 0.05 was considered as statistically significant.

## Results

Forty patients participated in this study, and the mean age of the intervention group was 43.60 ± 4.57 years. The minimum age among the patients in the intervention group was 32 years and that of the control group was 33 years. The maximum age in the both groups was 49 years. No significant difference was found between the two groups in terms of age (Log Rank test χ2, 1 degree of freedom = 6.81, P = 0.738).

During the study period, three patients (from intervention group) were excluded from the study due to experiencing medication side effects, and three persons from the control group due to dissuasion and lack of consent for continuing the study.

In this study, the mean of systolic and diastolic blood pressures was not significantly different between the two groups based on the results of the t- test (peripheral systolic blood pressure = 0.742) and (peripheral diastolic blood pressure = 0.796).

At the end of the study, systolic and diastolic blood pressures of both groups were decreased.

The mean of systolic blood pressure of the intervention group had a reduction of 40.86 mm Hg and that of the control group a reduction of 16 mm Hg. The mean of diastolic blood pressure of the intervention group had a reduction of 17.9 mm Hg and that of the control group a reduction of 6.7 mm Hg (Log Rank test χ2, 1 degree of freedom = 8.15, P<0.001). Blood pressure of both groups at the start and at the end of the study is demonstrated in Table 1.

The reduction of systolic and diastolic blood pressure was statistically significant after prescribing medication in the intervention group (P<0.001 for both). 

**Table1:** Blood Pressure of Intervention and Control Groups at the Start and End of Refractory Hypertension Study

	**Mean and standard deviation of** **diastolic blood pressure**			**Mean and standard deviation of systolic ** **blood pressure**		
	**At the start of ** **study**	**At the end of ** **study**	**P value**	**At the start of ** **study**	**At the end of ** **study**	**P value**
Intervention group	16/4 ±152/6	0/10 ± 7/111	P>0.001	6/5 ± 93/2	2/7 ± 3/75	P>0.001
Control group	147/31±12/6	4/16 ± 3/131	P>0.001	6/6 ± 93/2	9.7 ± 5/88	P>0.001

**Figure1:**
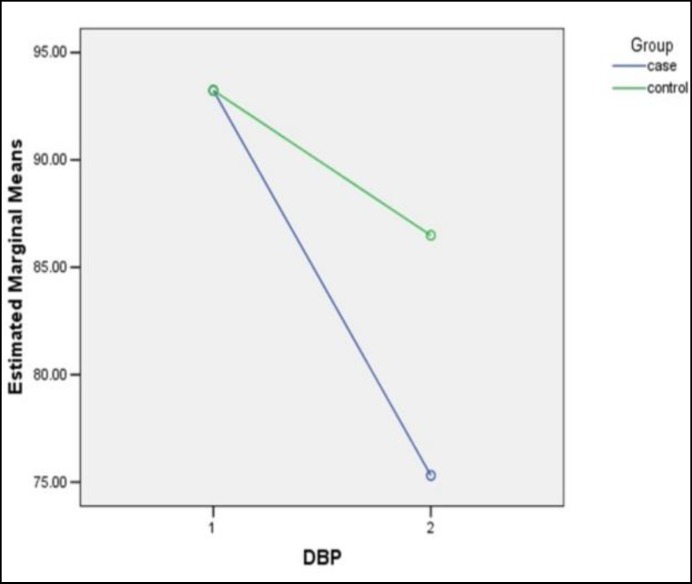
Mean Diastolic Blood Pressure in Both Groups at the Beginning and End of the Refractory Hypertension Study

In addition, reduction in systolic and diastolic blood pressure was statistically significant at the end of study in the control group (Log Rank test χ2, 1 degree of freedom = 10.52, P<0.001). The reduction rate of systolic blood pressure was not significantly different between the two groups before and after the intervention (Log Rank test χ2, 1 degree of freedom = 11.2, P = 0.11).

The reduction rate of diastolic blood pressure was statistically significant between the two groups before and after the intervention, and reduction of diastolic blood pressure was more in the intervention group (Log Rank test χ2, 1 degree of freedom = 4.88 , P=0.017). (Figure1). Effect size for the significant difference in the level of reduction in diastolic BP in intervention and control groups was respectively relative risk, 1.22; 95% CI, 0.94 to 1.50 and relative risk, 2.48; 95% CI, 1.85 to 3.11.

Placebo, compared to sertraline, had a lower effect on diastolic blood pressure of the patients (There was a statistically significant difference between the effect of placebo and sertraline on the reduction of diastolic blood pressure) (Log Rank test χ2, 1 degree of freedom = 6.24, P = 0.017)

In this study, the observed side effects in prescribing five-week sertraline with dose of 50 mg daily, which were clinically important for patients were nausea (45%), stomach ache (25%), drowsiness (20%), increase or decrease of appetite (20%), vomiting (15%) and feelings of inner tension (15%). No side effects were reported in placebo group.

Attention should be paid to the point that a major part of these side effects is almost clinically negligible and is seen in prescribing placebo. 

## Discussion

Since blood pressures of both groups were controlled by sphygmomanometer at a cardiology clinic, perhaps stress and anxiety resulted by measuring blood pressure on patient is more on systolic blood pressure rather than diastolic one. After the intervention, the systolic blood pressure reduced in the control group and it might have masked the probable effect of medication on systolic blood pressure.

In addition, in this study, basic blood pressure of both groups was measured by normal barometer, but blood pressure was registered and controlled by a 24-hour monitoring after the intervention. However, checking blood pressure at clinics could lead to momentary hypertension in some people (18).

In a case report study, hypertension in two women with recurrent hypertension after both were diagnosed with PMS, after treatment PMS problems with 25 mg amitriptyline and 1/5 mg Bromazepam daily during luteal phases per cycle in one case, and 50 mg sertraline daily and 1/5 mg Bromazepam twice a day, in the other one besides of antihypertensive drugs, blood pressure was well controlled. The authors suggested that in women with refractory hypertension who are not yet in menopause, PMS and the treatment beside of antihypertensive treatment should be considered (11).

In a study conducted on women with PMS, it was found that diastolic blood pressure (DBP), heart rate (HR) and (SBP) systolic blood pressure in women with PMS were higher in healthy subjects and the difference was statistically significant (19).

Considering that reduction in diastolic blood pressure in the group receiving PMS treatment (intervention group) was significantly different compared with that of the control group, it may be possible that accompaniment of PMS and HTN has higher effect on DBP rather than SBP. This issue should be investigated more in further studies.

In a study, plasma aldosterone level was higher in women with PMS during luteal phase compared with that of the control group.

Water and salt retention during luteal phase takes place in women with PMS (20); and one reason for hypertension in these people may be the retention of water and salt during luteal phase.

Some studies have shown that norepinephrine concentration in luteal phase in considerably higher in that in follicular phase (21, 22). Increase in norepinephrine concentration could be a reason for hypertension.

Some studies showed that activity of sympathetic nervous system (SNS) during luteal phase is more compared to during follicular phase (23-26).

Increase in the activity of SNS and decrease in the activity of parasympathetic nervous system during luteal phase could justify hypertension in people with PMS (27).

According to the findings of this study, it is suggested that in women with refractory hypertension who are not in their menopausal period, diagnosis and treatment of PMS be considered as a comorbid disease for a better control of their diastolic hypertension.

In terms of side effects of sertraline during the five weeks of prescription, some side effects of this study were more than those of the others; for instance, in this study nausea was reported to be 45%, which was 27% in another study (25). In this study, drowsiness was 20%, but it was 14% in the another study (28).

On the other hand, some of the reported side effects of this study were less than those of other studies. For example, in this study, significant sexual side effects were not reported. It should be mentioned that due to small sample size, low treatment period and type of consumed medication, generalization of medication side effects of this study or comparing them with the results of other studies is not possible.

## Limitations

In this study, recovery or failure in recovery in PMS symptoms of patients were not controlled, and the only criterion was prescription of medication. It is recommended to consider the control or lack of control of PMS symptoms and their effects on controlling patients’ blood pressure in the future studies.

## Conclusion

According to the findings of this study, prescribing 50 mg sertraline daily for women with refractory hypertension and comorbid PMS is more effective as an anti-hypertension medication and a better control of DBP than placebo and it is not due to its effect on anxiety conditions, since people with anxiety disorder were excluded of this study. This effect does not have a statistically significant difference with the effect of placebo about systolic blood pressure. Thus, further studies should be designed with higher populations or different subtypes of patients. It is suggested that further studies with larger sample size and with other common treatments of PMS be conducted in this field. In this study, considering the fact that sertraline was started for the trial group with a high dose, this may be the reason for high side effects reported in this study compared with other studies.
